# Initial Results of the International Efforts in Screening New Agents against *Candida auris*

**DOI:** 10.3390/jof8080771

**Published:** 2022-07-25

**Authors:** Vanice Rodrigues Poester, Lívia Silveira Munhoz, Jéssica Louise Benelli, Aryse Martins Melo, Abdullah M. S. Al-Hatmi, David J. Larwood, Marife Martinez, David A. Stevens, Melissa Orzechowski Xavier

**Affiliations:** 1 Programa de Pós-Graduação em Ciências da Saúde, Faculdade de Medicina (FAMED), Universidade Federal do Rio Grande (FURG), Rio Grande 96201-900, Brazil; vanicerp@gmail.com (V.R.P.); liviasmunhoz@gmail.com (L.S.M.); 2 Hospital Universitário Dr. Miguel Riet Corrêa Jr.-HU-FURG/Empresa Brasileira de Serviços Hospitalares—EBSERH, Rio Grande 96201-900, Brazil; jessicalb.jl@gmail.com; 3 National Institute of Health, Dr. Ricardo Jorge, 1649-016 Lisbon, Portugal; arysemartins@gmail.com; 4 Natural & Medical Science Research Center, University of Nizwa, Nizwa 616, Oman; abdullaalhatmi@gmail.com; 5 California Institute for Medical Research, San Jose, CA 95128, USA; davidl@valleyfeversolutions.com (D.J.L.); marifespiritu@yahoo.com (M.M.); stevens@stanford.edu (D.A.S.); 6 Valley Fever Solutions, Tucson, AZ 85719, USA; 7 Division of Infectious Diseases and Geographic Medicine, Stanford University Medical School, Stanford, CA 94305, USA

**Keywords:** *Candida auris*, *Candida* species, diphenyl diselenide, nikkomycin Z, antifungal drugs, organoselenium compounds, chitin synthase inhibitor, in vitro susceptibility assays

## Abstract

Background: *Candida auris* is an emergent fungal pathogen and a global concern, mostly due to its resistance to many currently available antifungal drugs. Objective: Thus, in response to this challenge, we evaluated the in vitro activity of potential new drugs, diphenyl diselenide (PhSe)_2_ and nikkomycin Z (nikZ), alone and in association with currently available antifungals (azoles, echinocandins, and polyenes) against *Candida auris*. Methods: Clinical isolates of *C. auris* were tested in vitro. (PhSe)_2_ and nikZ activities were tested alone and in combination with amphotericin B, fluconazole, or the echinocandins, micafungin and caspofungin. Results: (PhSe)_2_ alone was unable to inhibit *C. auris*, and antagonism or indifferent effects were observed in the combination of this compound with the antifungals tested. NikZ appeared not active alone either, but frequently acted cooperatively with conventional antifungals. Conclusion: Our data show that (PhSe)_2_ appears to not have a good potential to be a candidate in the development of new drugs to treat *C. auris*, but that nikZ is worthy of further study.

## 1. Introduction

*Candida auris* is an emergent fungal pathogen [1]. A global concern regarding this yeast is its misidentification, its resistance to many currently available antifungal drugs, virulence factors, high rates of spreading in hospital environments, and a high rate of mortality in the infected patients [2].

In response to this challenge, new effective options of antifungals are urgent. As a result, researchers in four countries (four continents) have come together in a consortium to perform an initial screening of unconventional agents that might prove useful, and discard those that may not indicate further efforts. This is the report of this work, which indicates the need for further and expanded studies.

Several entities have been tested against *C. auris* in vitro, such as nanoparticles, natural compounds, repurposing drugs, and other chemicals [3,4,5,6,7,8,9]. Of these drugs, ebselen, an organoselenium compound, is suggested to be a promising antifungal drug, owing to activity against several fungi [3]. It also appears to have in vitro activity against *C. auris* [4,5]. Diphenyl diselenide (PhSe)_2_ is another promising organoselenium compound with broad antifungal properties [3]. (PhSe)_2_ is a stable compound with minor toxicological effects in oral doses (30 μg), even after a long period of administration (8 months) [10,11]. Apparently, (PhSe)_2_ acts in the fungal cells interacting with sulfhydryl groups leading to a pro-oxidant effect to fungal cells associated with the glutathione (GSH) levels [12,13]. Therefore, considering the more advantageous properties of (PhSe)_2_ compared to ebselen [3], we selected (PhSe)_2_ in the initial screen.

Nikkomycin Z (nikZ) is an intriguing antifungal drug, as yet unlicensed in any country. It is of interest because of its unique mode of action, and because it appears to have an extremely low order of toxicity in vivo [14]. In addition, nikZ activity against other *Candida* species in vitro [15,16,17,18,19,20] and in vivo is known [15,19,21], including a report of in vitro activity against some *C. auris* isolates [22]. It also has a unique mode of action: it is structurally similar to UDP-N-acetylglucosamine, enabling it to be a competitive inhibitor for the enzyme chitin synthase, a fungal enzyme that processes UDP-N-acetylglucosamine to chitin, an important structural component in fungal cell walls [23]. This drug was selected also for the initial screen.

In this paper, we evaluate the in vitro activity of potential new drugs, (PhSe)_2_ and nikZ, alone and in association with currently available antifungals (azoles, echinocandins, and polyenes) against *Candida auris*.

## 2. Materials and Methods

Eleven clinical isolates of *C. auris* were included in the in vitro tests, ten from South Asian clade I and one from South Africa clade III (isolate # 20-247). All isolates were previously identified to species level [24]. (PhSe)_2_ was synthesized by the Paulmier [25] method through research collaboration with the Chemistry Department of the Federal University of Santa Maria (UFSM). NikZ was provided by Valley Fever Solutions, Tucson, AZ, USA. Amphotericin B (AmB) came from Cristália^®^ (Itapira, São Paulo, Brazil) or Abbott Laboratories^®^ (Research Park, Chicago, IL, USA), fluconazole (FCZ) from Sanobiol^®^ (Pouso Alegre, Minas Gerais, Brazil) or Pfizer^®^, Inc. (New York, NY, USA), and the echinocandins micafungin (MYC) came from Astellas^®^ Corp. (Tokyo, Japan) and caspofungin (CAS) from Merck^®^, Inc. (Whitehouse Station, NJ, USA).

Dilution assays were performed according to Clinical and Laboratory Standards Institute (CLSI) M27-Ed4 document [26]. All the MIC results were performed in triplicate, and the results never varied by more than one tube dilution; when there was this variation, the repeat results are presented in the tables. A susceptible internal control strain (*Candida parapsilosis*—CP90018 strain or *Candida kefyr*—SA strain) was included in each run as a quality control. Interactions between (PhSe)_2_ or nikkomycin Z and conventional antifungal drugs were evaluated by checkerboard assays [27], with the same inoculum as per CLSI methodology as for single drug testing, with incubation for 48 h at 35 °C in all tests. Under the conditions of our assays, with our isolates, growth at 24 h was not consistently adequate for assaying. The MIC was defined as the lowest concentration able to inhibit 100% ((PhSe)_2_, nikZ, AmB) or 50% (FCZ, CAS, MYC) of the fungal growth [26].

Drug concentrations tested, alone and in combination, ranged from 0.03 to 8 µg/mL for MYC, from 0.13 to 64 μg/mL for FCZ, from 0.03 to 16 μg/mL for AmB, from 0.19 to 50 for CAS [26], from 0.5 to 64 or 128 for nikZ [20,28,29], and from 0.5 to 32 µg/mL for (PhSe)_2_ [30]. We considered *C. auris* isolates to be resistant for the single drugs when MIC values were ≥32 µg/mL to FCZ, >2 µg/mL to AmB or CAS, and ≥4 µg/mL to MYC, as recommended [31], although as that guideline states: “There are currently no established *C. auris*-specific susceptibility breakpoints. Therefore, breakpoints are defined based on those established for closely related *Candida* species and on expert opinion. Correlation between microbiologic breakpoints and clinical outcomes is not known at this time”. The upper limits in our dilution series were either those recommended by the CLSI protocol or set well above what could be anticipated to be achieved in vivo, and we considered assaying higher concentrations to be irrelevant. Concentrations higher than 32 µg/mL for (PhSe)_2_ could not be achieved because of solubility issues.

A Fractional Inhibitory Concentration (FIC) for a drug in combination tubes is the result of dividing the MIC of the drug alone against a microbe, into that drug’s concentration in a combination tube showing inhibition. The Fractional Inhibitory Concentration Index (FICi) is determined by the sum of the FICs for both drugs, in the combination tube with the lowest sum of FICs [27]. We then classified the interaction as: strong synergism (SS) when FICi < 0.5, weak synergism (WS) when 0.5 < FICi < 1, additive (AD) when 1 < FICi < 2, indifferent (IND) when FICi = 2, or antagonistic (ANT) when FICi > 2 [32,33,34]. Classifications of FICi have been variable in the literature. Checkerboard tests were performed in duplicate (two independent experiments). For all tests, an internal control strain, as above, was used as a control of the antifungals’ potency.

## 3. Results

The total of assays in this study provides the data for 57 individual MIC determinations of (PhSe)_2_ and nikZ against *C. auris* clinical isolates, and 63 checkerboard studies of these drugs with commercial antifungals (each with the repetitions stated) (Table 1 and Table 2).

Part 1: (PhSe)_2_. (PhSe)_2_ was unable to inhibit 10 *C. auris* isolates even in the highest concentration tested (32 µg/mL) (Table 1). Almost all *C. auris* isolates appeared resistant to FCZ, and an occasional one resistant to AmB (Table 1 and Table 2). MICs of MYC were below the proposed cutoff, with MIC values ranging from 1 to 2 µg/mL (Table 1 and Table 2).

Only an additive inhibitory effect of (PhSe)_2_ was detected, and only with MYC against a minority of the isolates, 30% (*n* = 3). On the other hand, (PhSe)_2_ had an antagonistic effect against 100% of isolates when combined with AmB, as well as against one isolate with FCZ. All of the other interactions were indifferent (Table 1).

Part 2: nikZ (Table 2). NikZ was unable to inhibit *C. auris* isolates tested even in the highest concentration tested (64 or 128 µg/mL). The interaction with AmB did not show antagonism, but lacked strong synergy. For two isolates, strong synergy with FCZ was shown.

Given the more promising MYC results, it was then of interest to study another echinocandin, CAS. The MICs with CAS alone were more variable than the range seen with MYC (Table 1 and Table 2). There was impressive synergy, with four of six isolates, with combination nikZ-CAS. Interactions with the echinocandin CAS with nikZ were more positive, overall, than with the echinocandin MYC. Of interest, for two of the six isolates studied with CAS, the paradoxical effect (decreasing inhibition in some concentrations above the inhibitory endpoint) that we have described for other *Candida* species was noted [35], with CAS alone, and also in some inhibitory combinations with CAS; this phenomenon does not enter into the endpoint definitions here, as detailed elsewhere [35]. On the other hand, by the 50% definitions given for the CAS inhibitory endpoints, a synergistic result would be declared for a combination tube that merely showed ≥50% inhibition at a CAS concentration lower than the MIC for CAS (see the results in Table 2). However, for some of the combination tubes in the CAS checkerboard studies, the tube determining the FICi and some others actually achieved 100% inhibition, never seen with either CAS or nikZ alone here, suggesting an even more profound inhibitory interaction in some instances.

## 4. Discussion

The search for new drugs with anti-*C. auris* activity is urgent in view of the multi-drug resistance of this pathogen [1]. The two agents studied in this paper were particularly of interest in that (in contrast to echinocandins and polyenes) both are available for oral dosing. Drug interaction studies in “checkerboard” fashion, or other methodologies, is labor-intensive and reagent-consuming, but could point to additional avenues for combination therapy. Whatever limitations the present dataset may have, it is important here to canvas potential new agents against this pathogen, and to disseminate preliminary findings in a timely fashion. Our findings should be useful to suggest where further efforts should be directed and expanded. They also suggest unproductive future avenues.

(PhSe)_2_, similarly to ebselen, interacts with glutathione enzymes, which can provide antioxidant protection to host and a pro-oxidant prejudicial activity to fungal cells [12,36,37]. However, the exact mechanism of antifungal activity of these compounds is not completely understood. Although the in vitro activity of ebselen against *C. auris*, with MIC ranging from 0.5 to 8 µg/mL has been described [4,5], our study showed the inability of (PhSe)_2_ to inhibit *C. auris* in vitro in concentrations up to 32 µg/mL.

This difference between both organoselenium compounds activity could be attributed to the fact that ebselen can also target the proton pump H+-ATPase (Pma1p) in the fungal membrane, which is essential to fungal survival [38]. In addition, its molecule shows a ligation between a selenium and nitrogen atom, which is absent in the (PhSe)_2_ molecule, and this linkage could confer some antifungal activity [39]. However, antagonistic or indifferent effects predominated in the association of (PhSe)_2_ with presently available antifungals against *C. auris*. Unfortunately, although the potential of (PhSe)_2_ for future development of an antifungal drug has been proposed against other fungal pathogens [3], it seems that would not have an applicability to the emergent yeast *C. auris*.

In contrast, nikkomycin Z appears the more productive avenue for further studies. Our data suggest nikZ alone is not promising against this pathogen, although a prior study suggested a range of nikZ MICs [22]. The differences could be related to different isolates, different methodology, or different interpretations. The positive interactions, particularly with the echinocandin, and to a lesser extent the azole and the polyene AmB, indicates the need for more extensive such testing when circumstances permit, particularly finding the best echinocandin and azole for future drug interaction studies. There is extensive published data about the synergistic drug interactions of nikZ with azoles, and with echinocandins, against other fungal pathogens, both in vitro and in vivo [17,18,19,20,21,40,41,42]. The study of directed mutations, generated in the laboratory, in *C. auris* and involving drug mechanisms of action, could help to elucidate reasons for resistance with single drugs, and the efficacy or lack of efficacy in combinations. Aside from the limitations of limited datasets, it will also be important to study isolates from other clades of *C. auris*. Testing of combinations in vivo is also a critical need.

## 5. Conclusions

Our data show that (PhSe)_2_ appears to not have a good potential to be a candidate in the development of new drugs to treat *C. auris*, but that nikZ is worthy of further study, particularly in relation to the most effective combination therapy. Although the database in this paper on the echinocandins is yet small, there may be differences suggested between the echinocandins in the efficacy of interactions against this pathogen, and this should be a subject for further study.

## Figures and Tables

**Table 1:** Results of minimal inhibitory concentration (MIC) of alone drugs and fractional inhibitory concentration index (FICi) of in vitro interaction between diphenyl diselenide (PhSe)_2_ and micafungin (MYC), amphotericin B (AmB), or fluconazole (FCZ) against *Candida auris*.

Isolate #	MIC * (PhSe)_2_ Alone	MIC MYC Alone	FICi/INT ** (PhSe)_2_ + MYC	MIC AmB Alone	FICi/INT (PhSe)_2_ + AmB	MIC FCZ Alone	FICi/INT (PhSe)_2_ + FCZ
20-182	>32	1	2/IND	1-2	>2/ANT	>64	2/IND
20-184	>32	1-2	2/IND	1-2	>2/ANT	>64	2/IND
20-185	>32	2	≤1.5/AD	1-2	>2/ANT	>64	2/IND
20-187	>32	1	≤1.25/AD	2-4	>2/ANT	>64	2/IND
20-189	>32	1-2	2/IND	2-4	>2/ANT	>64	2/IND
20-193	>32	1-2	2/IND	2-4	>2/ANT	4	>2/ANT
20-195	>32	1-2	2/IND	2-4	>2/ANT	>64	2/IND
20-197	>32	2	≤1.5/AD	1-2	>2/ANT	>64	2/IND
20-198	>32	1-2	2/IND	2	>2/ANT	>64	2/IND
20-200	>32	1	2/IND	2-4	>2/ANT	>64	2/IND

Minimal inhibitory concentration in mcg/mL; dashed values show 1-tube variations in repeated runs; INT: Interaction. MIC * expressed as µg/mL. INT **: <0.5 strong synergism (SS); 0.5–<1 weak synergism (WS); 1–<2 additive (AD); 2 indifferent (IND); >2 antagonism (ANT).

**Table 2:** Results of the minimal inhibitory concentration (MIC) of alone drugs and fractional inhibitory concentration index (FICi) of in vitro interaction between nikkomycin Z (nikZ) and micafungin (MYC), caspofungin (CAS), amphotericin B (AmB), or fluconazole (FCZ) against *Candida auris*.

Isolate #	MIC * NikZ Alone	MIC MYC Alone	FICi/INT ** NikZ + MYC	MIC AmB Alone	FICi/INT NikZ + AmB	MIC FCZ Alone	FICi/INT NikZ + FCZ
20-182	>128	1	≤1.5/AD	1-2	≤0.51/WS	>64	2/IND
20-184	>64	1-2	ND	1-2	≤0.5/WS	>64	≤0.28/SS
20-185	>64	2	ND	1-2	≤0.5/WS	>64	≤0.28/SS
20-187	>64	1	≤1.5/AD	2-4	≤0.53/WS	>64	2/IND
20-189	>128	1-2	≤1.25/AD	2-4	2/IND	>64	2/IND
20-193	>64	1-2	≤1.25/AD	2-4	≤0.5/WS	4	≤1/WS
20-195	>64	1-2	≤1/WS	2-4	≤0.5/WS	>64	2/IND
20-197	>64	2	ND	1-2	≤0.63/WS	>64	2/IND
20-198	>64	1-2	≤1.5/AD	2	≤0.5/WS	>64	2/IND
20-200	>64	1	≤1.25/AD	2-4	≤0.5/WS	>64	2/IND
		MIC CAS alone	FICi/INT ** NikZ + CAS				
20-182	>128	0.39	≤0.5/WS				
20-184	>64	25	≤0.25/SS				
20-185	>64	25	≤0.05/SS				
20-189	>128	0.39	≤0.5/WS				
20-197	>64	6.25	≤0.38/SS				
20-247	>128	3.13	≤0.27/SS				

Minimal inhibitory concentration in mcg/mL; dashed values show 1-tube variations in repeated runs; INT: Interaction. MIC * expressed as µg/mL. INT **: <0.5 strong synergism (SS); 0.5–<1 weak synergism (WS); 1–<2 additive (AD); 2 indifferent (IND); >2 antagonism (ANT). ND, not done.

## Data Availability

Not applicable.
